# Trends in Use and Expenditures for Brand-name Statins After Introduction of Generic Statins in the US, 2002-2018

**DOI:** 10.1001/jamanetworkopen.2021.35371

**Published:** 2021-11-22

**Authors:** Shuo-yu Lin, Kyle Baumann, Chenxuan Zhou, Weiyu Zhou, Alison Evans Cuellar, Hong Xue

**Affiliations:** 1 Department of Health Administration and Policy, College of Health and Human Services, George Mason University, Fairfax, Virginia; 2 Department of Medicine, University of Minnesota Medical School, Minneapolis; 3 Department of Pediatrics, University of Minnesota Medical School, Minneapolis; 4 School of Medicine, Virginia Commonwealth University, Richmond; 5 Department of Statistics, Volgenau School of Engineering, George Mason University, Fairfax, Virginia

## Abstract

**Importance:**

The high and increasing expenditures for prescription medications in the US is a national problem.

**Objective:**

To explore the association of generic statin competition on relevant use and cost savings and to provide use and expenditure trends for all available statins for private and public payers and for out-of-pocket spending.

**Design, Setting, and Participants:**

This survey study evaluated data from the January 1, 2002, to December 31, 2018, Medical Expenditure Panel Survey by using a difference-in-differences analysis. Participants included noninstitutionalized individual statin users. Data were analyzed from November 1, 2020, to March 30, 2021.

**Exposures:**

The market entry of 5 generic statin medications (atorvastatin, rosuvastatin, simvastatin, lovastatin, and pravastatin).

**Main Outcomes and Measures:**

National- and individual-level reductions in the annual number of statin purchases and total expenditures across private insurance, public insurance (Medicaid and Medicare), and out-of-pocket spending (presented in 2018 US dollars).

**Results:**

Between January 1, 2002, and December 31, 2018, an average of 21.35 million statins (95% CI, 16.7-25.5 million) were purchased annually, with an average total annual cost of $24.5 billion (95% CI, $18.2-$28.8 billion). The number of brand-name statin purchases decreased by 90.9% (95% CI, 56%-98%) nationally and 27.4% (95% CI, 13%-40%) individually after the end of market exclusivity. Among major payers, the end of market exclusivity was associated with individual cost savings of $370.00 (95% CI, $430.70-$309.20) for private insurers, $281.00 (95% CI, $346.80-$215.30) for Medicare, $72.34 (95% CI, $95.22-$49.46) for Medicaid, and $211.90 (95% CI, $231.20-$192.50) for out-of-pocket spending. Combining all payers, the decrease translates to $925.60 (95% CI, $1005.00-$846.40) of annual savings per individual and $11.9 billion (95% CI, $10.9-$13.0 billion) for the US.

**Conclusions and Relevance:**

Results of this survey study suggest that full generic competition of statins was associated with significant cost savings across all major payers within the US health care system.

## Key Points

**Question:**

How did expenditures for statins change after market exclusivity ended and generic statins became available?

**Findings:**

Using 17 years of the Medical Expenditure Panel Study data, this survey study of generic competition among statins found that the end of market exclusivity was associated with $925.60 of annual savings per individual and $11.9 billion in savings for the US.

**Meaning:**

Full generic competition of statins was associated with significant cost savings across all major payers within the US health care system.

## Teaser

This survey study investigates trends in statin use and expenditures after market exclusivity ended and generic statins became available in the US.

## Introduction

The high and increasing health care expenditure in the US is a national problem. In 2019 alone, national health care spending increased 4.6%.^1^ Retail prescription drug spending reached $369.7 billion (10% of total health care spending),^1^ resulting in immense public outcry.^2^ Improving the affordability of prescription drugs has become a focus of both the scientific and policy communities.^3,4^

Generic competition is one important policy tool to stem rising prescription drug expenditures.^3^ In the US, the entry of generic drugs into the market typically starts after the market exclusivity period of a brand-name drug ends.^4^ A generic drug is a pharmaceutically equivalent alternative to a brand-name drug that is made by another manufacturer and is sold at a fraction of the cost.^7,8^ According to the Center of Drug Evaluation and Research estimates, the average generic drug price is 49% of the corresponding brand-name drug.^9^ When 6 or more generic competitors exist, the reduction is up to 95%.^5^

Statins are a class of lipid-lowering medication used to prevent and treat atherosclerotic cardiovascular disease (ASCVD).^6^ In the US, heart disease is the leading cause of death for men and women across most racial and ethnic groups.^7^ From 2014 to 2015, heart disease resulted in $351.3 billion in annual direct and indirect costs.^8^ Per the American Heart Association guidelines, statin therapy has become the pharmaceutical cornerstone of ASCVD prevention and treatment, with an emphasis on high-intensity statin use for patients with clinical ASCVD or high ASCVD risk.^6^

The 7 available statins are classified into 3 levels of intensity (low, moderate, and high) based on pharmacologic potency and dosage.^9^ Before the end of their market exclusivity, Zocor (simvastatin) and Lipitor (atorvastatin) were 2 of the top-selling medicines in the world.^10^ Furthermore, Crestor (rosuvastatin) was 1 of the 3 most costly drugs for the Medicare Part D program in 2015.^11^ Between 2002 and 2013, the overall rate statin use among adults older than 40 years increased from 17.9% to 27.8%, with a total cost amounting to $16.9 billion in 2012-2013.^12^ As of 2021, the market exclusivity period of the 7 brand-name statins has expired, including Zocor (simvastatin) in 2006, Lipitor (atorvastatin) in 2011, and Crestor (rosuvastatin) in 2016.

Prior studies^21,35^ have shown that the introduction of generic competition was associated with (1) the reduction of Lipitor prescriptions in a large private insurer and (2) a large increase in the number of prescriptions filled for generic simvastatin in statewide Medicaid programs. Nevertheless, to our knowledge, the economic impact of generic competition for all available statins has not been comprehensively examined. In this survey study, we examine use and expenditure trends for all available statins, using data obtained on private health insurance, public health insurance (ie, Medicaid and Medicare), and out-of-pocket (OOP) spending. In addition, we examine the association between generic statin competition and relevant use and cost savings.

## Methods

### Data

A 17-year panel survey study was performed using Medical Expenditure Panel Survey data. The Medical Expenditure Panel Survey is an annual nationwide representative survey of the noninstitutionalized US population. In this study, we included data from January 1, 2002, to December 31, 2018. Full-year consolidated data and prescribed medicine files were merged to construct the analytical data sets.^13^ The Medical Expenditure Panel Survey is a publicly available and deidentified data file; per the US Department of Health and Human Services guidelines, this study was exempted from institutional review broad approval. Written consent was obtained from survey participants who were contacted for interviews and to contact their particular clinicians and pharmacies. This analysis and report followed the American Association for Public Opinion Research (AAPOR) 12-item reporting guideline (eTable 4 in the Supplement).

### Study Design

A panel difference-in-differences design was used to evaluate the outcome of end of market exclusivity on the annual number of brand-name generic statin purchases and expenditures. The first difference refers to the difference in the number of purchases and expenditures of each brand-name statin before and after the end of market exclusivity. The second difference refers to the difference between a given brand-name statin that ends market exclusivity in a given year and all other brand-name statins with market exclusivity yet to end at that time. This same design was used to analyze the generic statins (eAppendix 1 in the Supplement). Seven brand-name statins and the 5 generic statins available as of 2018 were included in the study. The preperiod trends were tested and determined to meet the parallel trends assumptions (eFigures 5-8 in the Supplement).

### Data Processes

Both national- and individual-level analyses were performed. We merged prescription drug event files with full-year consolidation files to obtain the number of statin purchases and corresponding expenditures at the individual level (n = 58 354) (eTable 3 in the Supplement). We then converted individual-level data to national-level data by aggregating the annual number of purchases and expenditures by each statin class. A final analytical panel-data structure with nationally representative estimates was then created based on statin class (n = 204). More detailed information about the data processes and sample selection is presented in eAppendix 1 and eFigure 1 in the Supplement.

### Annual Number of Statin Purchases and Expenditures

The main outcome variables were annual number of statin purchases and expenditures. Expenditures referred to what was paid for statin purchases by payers and individuals combined. We calculated total annual expenditure as well as mean payments by private insurance, Medicaid, Medicare, and OOP spending. The expenditure estimates were presented in 2018 US dollars (USD) based on Consumer Price Index prescribed medicines (ie, the price index for prescription drugs) (eAppendix 1 in the Supplement).^14^

### Key Independent Variable

The Multum Lexicon provided by the Agency for Healthcare Research and Quality was used to identify the class of statin.^15^ The level-1 drug therapeutic category of 358 identified metabolic agents, the level-2 code of 19 specified antihyperlipidemic agents, and the level-3 code of 173 classified 3-hydroxy-3-methylglutaryl coenzymes (HMG-CoA reductase inhibitors). The recorded drug name was used to specify whether a statin was branded or generic.

### Covariates

Age, sex (male and female), race and ethnicity (self-reported as Asian, Black or African American, and White), health insurance coverage (whether a person had private or public health insurance, or did not have any kind of coverage in the past year), usual source of care (defined as whether participants had a particular medical professional, doctor's office, clinic, or other place where they would usually go if sick or in need of advice about health), marital status (currently married or otherwise), geographic region (Northeast, Midwest, South, and West), and cardiovascular disease (ie, angina, congenital heart disease, stroke, and myocardial infarction) were included in the regression analysis.^6^ For the analysis at the national level, all covariates were presented as a percentage, other than age, which was presented as a mean.

### Statistical Analysis

Descriptive analysis was conducted to examine the temporal trend of purchases and expenditures in the study period from January 1, 2002, to December 31, 2018. A negative binomial model was developed to determine the annual number of statin purchases. Using γ distribution with log link, a generalized linear model was developed to estimate national-level annual expenditures. To estimate models of individual-level expenditures, which have a large number of zeros, we used a 2-part model—first part logit and second part generalized linear model.^16,17,18^ The modified Park test was performed to confirm the choice of γ family as appropriate (eTable 1 in the Supplement). Sampling weights and strata were implemented to generate nationally representative estimates (eAppendix 1 in the Supplement). A 2-sided *P* value <.05 was considered statistically significant. All statistical analyses were conducted from November 1, 2020, to March 30, 2021, using SAS, version 9.4 (SAS Institute Inc) and STATA, version 16.1 (StataCorp LLC).

## Results

### Summary Statistics

Between 2002 and 2018, an annual mean of 21.35 million (95% CI, 16.7-25.5 million) statin prescriptions were purchased nationally, with an average total annual cost of $24.5 billion (95% CI, $18.2-$28.8 billion). Private health care insurance and OOP spending accounted for 31.8% ($7.81 billion) and 25.5% ($6.25 billion) of statin expenditures, respectively, whereas Medicare accounted for 21.9% ($5.38 billion) and Medicaid for 5.3% ($1.3 billion) (Table 1). The mean (SE) age of statin users across the entire sample was 63.5 (0.34) years (95% CI, 62.78-64.11). Across the entire sample, 48% (SE, 0.01) statin users were female, 52% (SE, 0.01) were male, 4% (SE, 0.01) were Asian, 12% (SE, 0.01) were Black or African American, and 84% (SE, 0.01) were White. A total of 95% of the participants (SE, 0.01) had usual source of care. Sixty-five percent of statin users (SE, 0.01) were covered by private health insurance, 31% (SE, 0.01) by public health insurance, and 4% (SE, 0.01) were uninsured. A total of 62% of participants (SE, 0.01) were married; 17% of statins users (SE, 0.01) resided in Northeast, 24% (SE, 0.01) in the Midwest, 40% (SE, 0.01) in the South, and 19% (SE, 0.01) in the West.

**Table 1.** Weighted Summary Statistics of Annual Statin Use and Expenditures, 2002-2018

Statin	Lipitor	Atorvastatin	Zocor	Simvastatin	Mevacor	Lovastatin	Pravachol	Pravastatin	Lescol	Livalo	Crestor	Rosuvastatin
Market exclusivity period	1997-2011	Generic	1988-2006	Generic	1987-2001	Generic	1991-2006	Generic	2000-2020	2009-2020	2003-2016	Generic
Mean (SE) No. of purchases, million	4.25 (0.85)	3.37 (1.07)	1.14 (0.37)	5.70 (0.88)	0.03 (0.01)	1.36 (0.12)	0.47 (0.16)	1.78 (0.32)	0.19 (0.06)	0.02 (0.01)	1.58 (0.24)	0.28 (0.16)
Out-of-pocket expenditure, mean (SE) USD, billion	2.58 (0.57)	0.34 (0.12)	0.86 (0.32)	0.63 (0.11)	0.01 (0.01)	0.23 (0.04)	0.35 (0.13)	0.20 (0.03)	0.11 (0.03)	0.01 (0.01)	0.88 (0.15)	0.05 (0.02)
Medicare expenditure, mean (SE) USD, billion	1.46 (0.37)	0.89 (0.27)	0.21 (0.08)	0.78 (0.14)	0.01 (0.01)	0.20 (0.04)	0.10 (0.04)	0.35 (0.07)	0.03 (0.01)	0.02 (0.01)	1.20 (0.27)	0.14 (0.09)
Medicaid expenditure, mean (SE) USD, billion	0.49 (0.11)	0.16 (0.05)	0.16 (0.07)	0.12 (0.02)	0.01 (0.01)	0.03 (0.01)	0.10 (0.04)	0.04 (0.01)	0.01 (0.01)	0.01 (0.01)	0.19 (0.06)	0.01 (0.01)
Private insurance expenditure, mean (SE) USD, billion	3.06 (0.56)	0.72 (0.21)	0.77 (0.28)	0.72 (0.17)	0.02 (0.01)	0.20 (0.05)	0.35 (0.12)	0.20 (0.03)	0.06 (0.02)	0.02 (0.01)	1.54 (0.28)	0.15 (0.10)
Total annual expenditure, mean (SE) USD, billion	7.94 (1.44)	2.27 (0.66)	2.19 (0.77)	2.83 (0.40)	0.04 (0.01)	0.73 (0.14)	0.94 (0.32)	0.83 (0.14)	0.23 (0.06)	0.06 (0.03)	4.25 (0.73)	0.37 (0.22)
Past medical history, mean (SE) %												
Angina	0.09 (0.01)	0.14 (0.03)	0.10 (0.01)	0.11 (0.01)	0.10 (0.02)	0.08 (0.01)	0.09 (0.02)	0.07 (0.01)	0.08 (0.02)	0.07 (0.01)	0.09 (0.01)	0.19 (0.06)
Congenital heart disease	0.21 (0.01)	0.28 (0.05)	0.18 (0.02)	0.23 (0.02)	0.23 (0.06)	0.15 (0.01)	0.16 (0.02)	0.18 (0.02)	0.18 (0.03)	0.18 (0.02)	0.22 (0.02)	0.33 (0.06)
Myocardial infarction	0.15 (0.01)	0.21 (0.04)	0.14 (0.01)	0.17 (0.01)	0.10 (0.02)	0.12 (0.01)	0.12 (0.02)	0.12 (0.01)	0.11 (0.02)	0.13 (0.01)	0.14 (0.01)	0.21 (0.04)
Stroke	0.10 (0.01)	0.1 (0.03)	0.12 (0.03)	0.13 (0.01)	0.09 (0.02)	0.10 (0.01)	0.08 (0.01)	0.10 (0.01)	0.11 (0.01)	0.08 (0.01)	0.10 (0.01)	0.15 (0.05)
Access to care, mean (SE), %												
Having usual source of care	0.95 (0.01)	0.97 (0.01)	0.96 (0.01)	0.97 (0.01)	0.96 (0.01)	0.96 (0.01)	0.91 (0.06)	0.97 (0.01)	0.98 (0.01)	0.95 (0.01)	0.96 (0.01)	0.94 (0.03)
Private health insurance	0.65 (0.03)	0.60 (0.06)	0.68 (0.05)	0.61 (0.01)	0.66 (0.05)	0.57 (0.01)	0.72 (0.04)	0.57 (0.04)	0.65 (0.02)	0.62 (0.03)	0.70 (0.02)	0.65 (0.05)
Public health insurance	0.32 (0.03)	0.35 (0.05)	0.23 (0.02)	0.36 (0.01)	0.31 (0.04)	0.39 (0.01)	0.25 (0.03)	0.39 (0.04)	0.32 (0.02)	0.34 (0.03)	0.27 (0.02)	0.27 (0.05)
No health insurance	0.03 (0.01)	0.05 (0.02)	0.09 (0.06)	0.04 (0.01)	0.03 (0.01)	0.04 (0.01)	0.03 (0.01)	0.04 (0.01)	0.03 (0.01)	0.04 (0.01)	0.02 (0.01)	0.08 (0.03)
Married, mean (SE) %	0.63 (0.02)	0.59 (0.04)	0.65 (0.03)	0.61 (0.01)	0.66 (0.05)	0.56 (0.01)	0.59 (0.05)	0.58 (0.01)	0.60 (0.01)	0.62 (0.01)	0.64 (0.01)	0.63 (0.06)
Race and ethnicity, mean (SE) %												
Asian	0.05 (0.01)	0.05 (0.01)	0.03 (0.01)	0.03 (0.01)	0.09 (0.03)	0.06 (0.01)	0.03 (0.01)	0.03 (0.01)	0.03 (0.01)	0.04 (0.01)	0.04 (0.01)	0.02 (0.01)
Black or African American	0.10 (0.01)	0.11 (0.03)	0.14 (0.05)	0.10 (0.01)	0.13 (0.06)	0.09 (0.01)	0.08 (0.01)	0.13 (0.03)	0.13 (0.01)	0.10 (0.01)	0.08 (0.01)	0.17 (0.05)
White	0.85 (0.02)	0.84 (0.03)	0.82 (0.05)	0.85 (0.01)	0.78 (0.05)	0.84 (0.01)	0.89 (0.02)	0.83 (0.02)	0.84 (0.01)	0.87 (0.01)	0.88 (0.01)	0.81 (0.05)
Sex, mean (SE) %												
Female	0.46 (0.02)	0.35 (0.05)	0.41 (0.04)	0.40 (0.04)	0.54 (0.06)	0.50 (0.01)	0.57 (0.06)	0.51 (0.01)	0.60 (0.03)	0.58 (0.04)	0.50 (0.02)	0.29 (0.05)
Male	0.65 (0.54)	0.50 (0.65)	0.40 (0.59)	0.54 (0.60)	0.42 (0.46)	0.50 (0.01)	0.46 (0.43)	0.43 (0.49)	0.49 (0.4)	0.71 (0.42)	0.60 (0.5)	0.59 (0.71)
Age, mean (SE), y	63.38 (0.65)	63.91 (1.59)	62.32 (0.92)	65.19 (0.44)	64.12 (0.66)	65.00 (0.37)	65.04 (1.49)	61.17 (2.11)	65.93 (1.02)	62.39 (0.71)	61.65 (0.55)	61.57 (2.07)

Abbreviation: USD, US dollars.

### Trends of Statin Use and Expenditures

**Figure 1.**
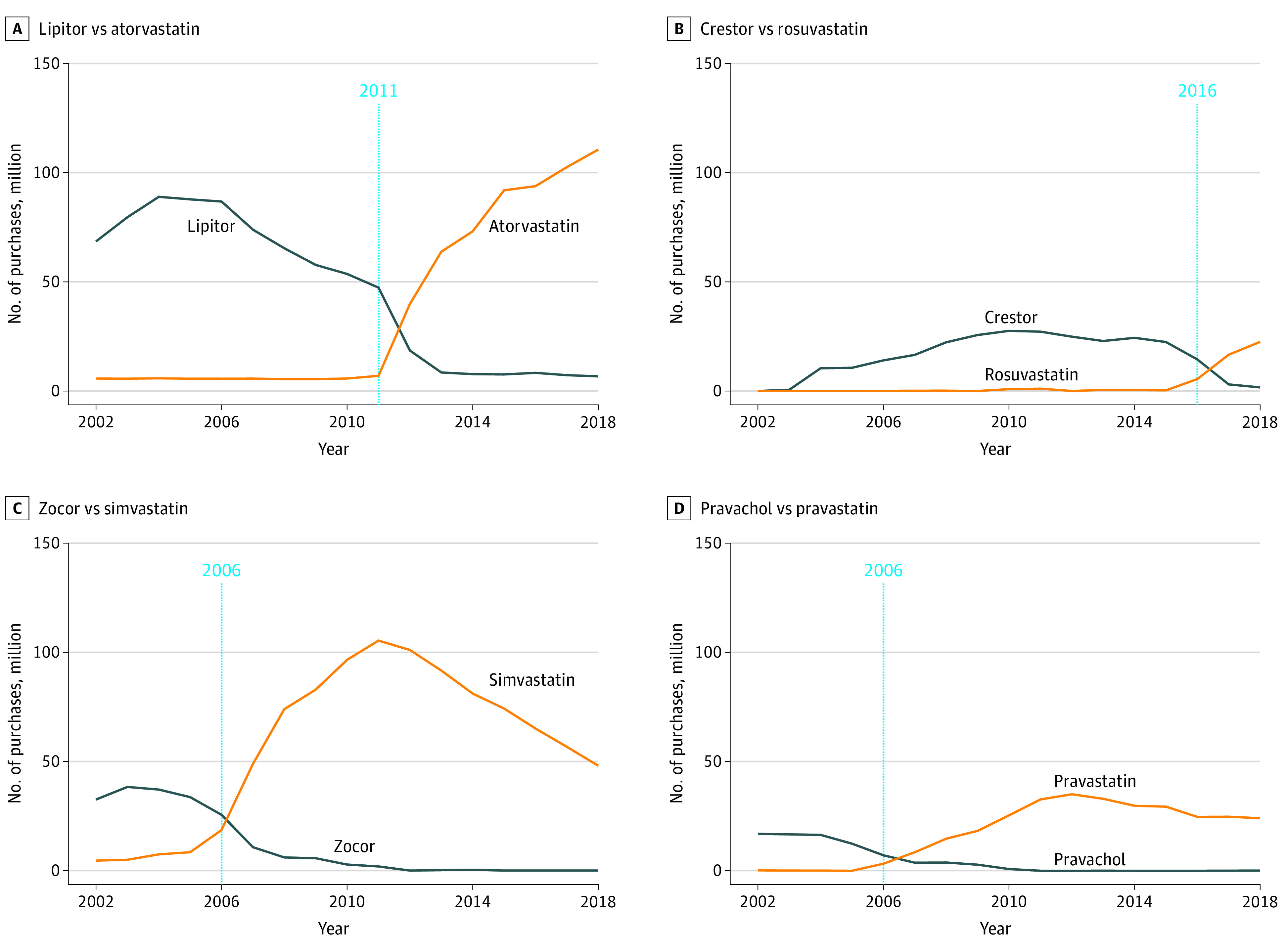
Trends in Selected Statin Use Before and After End of Market Exclusivity Each panel shows the number of purchases for a specific brand-name statin vs its generic equivalent.

**Figure 2.**
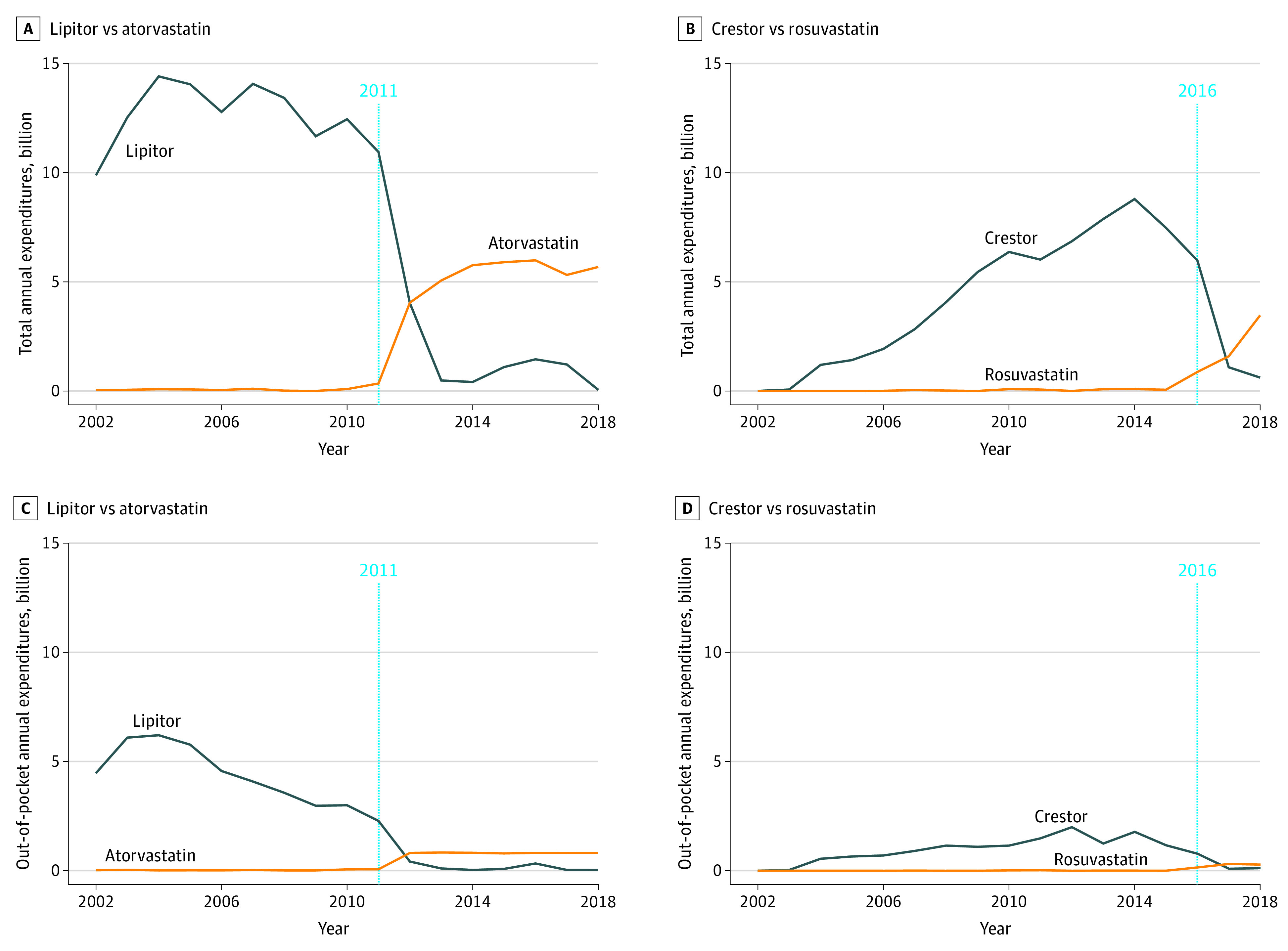
Trends of Annual Total and Out-of-pocket Expenditures in Most Prescribed Statins Before and After the End of Market Exclusivity Each panel shows annual total expenditures for a specific brand-name statin vs its generic equivalent. The dotted blue line in each panel indicates the end of market exclusivity.

After the end of market exclusivity, the number of brand-name statin purchases decreased across branded statins (Figure 1). For instance, the annual prescriptions of Crestor surged to 24.8 million in 2015, just 1 year before the end of exclusivity, with national spending peaking at $8.79 billion in 2014. The number of Crestor purchases dropped to 1.63 million in 2018 with $0.61 billion in annual expenditures (Figure 2). During the same period, the number of generic rosuvastatin purchases exceeded Crestor, reaching 22.5 million purchases in 2018. The detailed trend analysis for atorvastatin and simvastatin are in eAppendix 2 in the Supplement.

**Figure 3.**
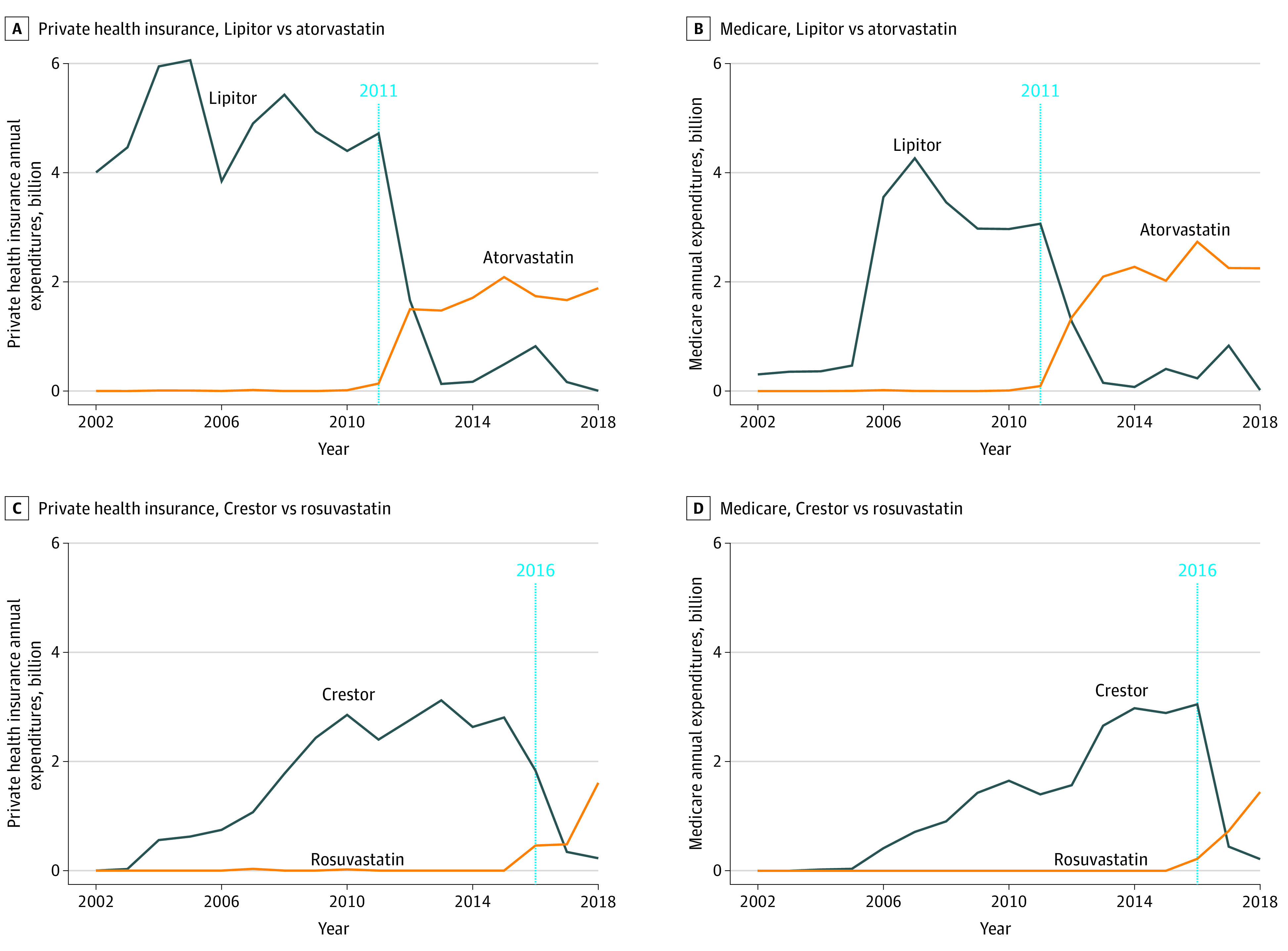
Trends of Insurer Spending for High-Intensity Statins Before and After the End of Market Exclusivity Trends of private health insurers (A) and Medicare (B) for Lipitor vs atorvastatin. Trends of private health insurers (C) and Medicare (D) for Crestor vs rosuvastatin before and after end of market exclusivity (dotted blue line).

Examining changes by drug intensity, the results suggest that the most prescribed statins, namely high-intensity statins, exhibited the greatest decline in expenditures across private health insurance, Medicare, Medicaid, and OOP spending (Figure 3; eFigure 4 in the Supplement). Private insurance spending for Lipitor fell from $4.72 billion to $0.13 billion 2 years after the end of market exclusivity. Following a rebound to $0.82 billion in 2016, total spending dropped to $0.01 billion by 2018. Annual Medicare expenditures for high-intensity statins fell sharply to $0.15 billion in 2013 and $0.02 billion in 2018. Annual spending by Medicare and private health insurance for generic atorvastatin rose to $2.25 billion and $1.88 billion, respectively, in 2018. Notably, Lipitor’s OOP expenditure began to decline even before the end of market exclusivity, dropping to $0.41 billion. Trends for other statins can be found in eFigure 2 in the Supplement.

Spending on Crestor showed great decrease across all major payers. Since its approval in 2003, annual private insurance expenditures for Crestor quintupled over 10 years from $0.56 to $3.12 billion and quadrupled from $0.41 billion in 2006 to $3.05 billion in 2016 after the Medicare Part D launch of 2006. However, after market exclusivity ended in 2016, annual expenditures for private insurance and Medicare fell to $0.23 billion and $0.21 billion, respectively, in 2018. In contrast, private insurance spending on generic rosuvastatin was stable in 2016 to 2017, followed by a surge to $1.61 billion by the end of 2018. Medicaid and OOP expenses for statins showed a similar pattern (Figure 2; eFigure 4 in the Supplement). Detailed trend analysis of low-intensity statins can be found in eFigure 3 in the Supplement.

### Association Between End of Market Exclusivity and Statin Purchases and Expenditure

The end of market exclusivity was associated with substantial cost savings for individuals and the US as a nation. Nationally, the number of brand-name statin purchases decreased by 90.9% (95% CI, 56%-98%) following the end of market exclusivity (Table 2). Individually, a 27.4% (95% CI, 13%-40%) decrease in the number of brand-name statin purchases was associated with the end of market exclusivity (Table 2). At both the national and individual level, market exclusivity was associated with significant expenditure reductions for brand-name statins: 62% for private insurers ($370.00 USD decrease individually; 95% CI, $430.70-$309.20), 70.1% for Medicaid ($72.34 USD decrease individually; 95% CI, $95.22-$49.46), 98.6% for Medicare ($281.00 USD decrease individually; 95% CI, $346.80-$215.30), and 71.3% ($211.90 USD decrease individually; 95% CI, $231.20-$192.50) for OOP spending after the end of market exclusivity (Table 2). Combining all payers, generic competition was associated with a 90.9% (95% CI, 56%-98%) reduction in national annual expenditure for statins. This decrease translates to $925.60 (95% CI, $1005.00-$846.40) of annual savings per individual and $11.9 billion (95% CI, $10.9-$13.0 billion) for the US. Subgroup analyses for primary and secondary prevention indications and various racial and ethnic groups are presented in eTable 2 in the Supplement.

**Table 2.** Impact of End of Market Exclusivity on Number of Statin Purchases and Expenditures per Panel Difference-in-Differences Estimates

Variable	No. of purchases, IRR (95% CI)^b^	Estimated effects^a^ (95% CI)				
		Private insurance^c^^,^^d^	Medicare^c^^,^^d^	Medicaid^c^^,^^d^	Out of pocket^c^^,^^d^	Total annual expenditure^c^^,^^d^
National level						
Brand name	0.09^e^ (0.02 to 0.44)	−0.96 (−3.45 to 1.51)	−4.23^f^ (−9.05 to 0.57)	−1.21 (−3.95 to 1.55)	−1.25 (−3.50 to 1.01)	−2.12 (−4.67 to 0.41)
Interpretation %	90.90 Decrease	62.00 Decrease	98.60 Decrease	70.10 Decrease	71.30 Decrease	88.10 Decrease
Generic	3.13 (0.52 to 18.84)	3.36^e^ (1.19 to 5.53)	2.55^g^ (0.08 to 5.01)	0.08 (−2.05 to 2.22)	1.53 (−0.56 to 3.62)	1.22 (−0.84 to 3.28)
Interpretation	2.10 Times increase	27.00 Times increase	11.80 Times increase	9% Increase	3.60 Times increase	2.38 Times increase
Individual level						
Brand name	0.73^e^ (0.60 to 0.87)	−370.00^h^^,^^e^ (−430.70 to −309.20)	−281.00^e^ (−346.80 to −215.30)	−72.34^e^ (−95.22 to −49.46)	−211.90^e^ (−231.20 to −192.50)	−925.60^e^ (−1005.00 to −846.40)
Interpretation	27.40% Decrease	$370.00 Reduction	$281.00 Reduction	$72.34 Reduction	$211.90 Reduction	$925.60 Reduction
Annual expenditure saved by end of market exclusivity	NA	NA	NA	NA	NA	11.9 Billion (10.9 to 13.0 billion)

Abbreviations: IRR, incidence rate ratio; NA, not applicable.

^a^ Estimated effects, at the national level, refer to the change of expenditures after the end of market exclusivity in comparison with the prior period, estimated from regression models. At the individual level, the effect estimates refer to the regression estimates of cost saving for each individual after the end of market exclusivity compared with the prior period.

^b^ No. of purchases (presented in IRR) was estimated by negative binomial model, controlling for female sex; Asian, Black, and White race; marital status; public health insurance; usual source of care; past medical history (ie, stroke, heart attack, congenital heart disease, angina); and age.

^c^ National-level expenditure was estimated by generalized linear model, γ distribution with log link function, controlling percent of female sex; Asian, Black, and White race; marital status; public health insurance; usual source of care; past medical history (ie, stroke, heart attack, congenital heart disease, angina); and average age.

^d^ Individual-level expenditure was estimated by 2-part model, with first part logit and second part generalized linear model following γ distribution with log link function, controlling for female sex; Asian, Black, and White race; marital status; public health insurance; usual source of care; past medical history (ie, stroke, heart attack, congenital heart disease, angina); and age.

^e^ *P* < .01.

^f^ *P* < .10.

^g^ *P* < .05.

^h^ The cost savings for private insurance was greater than other payers (Medicaid, Medicare, and out of pocket, *F* score = 11.79; *P* < .01).

## Discussion

In this survey study, we analyzed the use and cost trends of all available statins in the US and evaluated the outcome of generic competition on expenditures, at both the national and individual levels. Overall, the introduction of generic statins was associated with a 90.9% reduction in number of purchases on brand-name statins at the national level ($925.60 USD of cost savings for an individual) and a 2.1-times increase in the generic statins purchases. Although experienced by all, the magnitude of benefit varied between spenders. Private insurance experienced the greatest savings, followed by Medicare, OOP spenders, and Medicaid. Overall, $11.9 billion in savings were associated with the introduction of generic competition.

### The Benefits of Generic Competition

Generic competition is considered the most effective tool for reducing prescription prices in the US.^19^ In 2017, although accounting for just 10% of total prescriptions, brand-name drugs represented 77% of the spending; the 90% of generic prescriptions generated $265 billion in savings, with Medicare and Medicaid savings amounted to $82.7 billion and $40.6 billion, respectively.^20^ The savings translated to an average $1952.00 for each Medicare enrollee and $568.00 for each Medicaid enrollee.^20^ Using the saving estimates from the previous report, we estimated that 14.4% ($281.00 of $1952.00) of Medicare savings and 12.7% ($72.34 of $568.00) of Medicaid savings could have been generated from generic statins.

In line with the findings of existing studies on high blood pressure medications such as generic substitution of angiotensin^1^ and on the substitution of generic oral contraceptives,^2^ the current study found that generic entry was associated with a great reduction of brand-name statin purchases. This reduction will likely continue to grow, especially for high-intensity statins. Following the release of the 2018 American College of Cardiology/American Heart Association Guideline on the Management of Blood Cholesterol recommendations,^6,9^ high-intensity statins (ie, atorvastatin and rosuvastatin) became the most dominant statin class in the US, accounting for 62.83% of the market share in 2018 (Table 1). As shown in prior studies,^21,22^ full generic competition (defined as having 4 or more generic manufactures for a certain statin) reduced brand-name atorvastatin prescriptions significantly within 1 large private health insurance company and saved the US health care system approximately $1.9 billion. Our results indicate that full generic competition of all statins occurred within 1 to 2 years and resulted in an 88.1% reduction in total annual brand-name statin expenditures.

Comparable reductions were also observed for the low- to moderate-intensity statins. Sales of Zocor declined dramatically after the end of market exclusivity.^23^ Our data confirmed this and further demonstrated a surge in low- to moderate-intensity statin purchases between 2007 and 2012, with a concurrent moderate increase in total annual expenditures. For the least-prescribed, low- to moderate-intensity statins (ie, Lescol [fluvastatin] and Livalo [pitavastatin]), we estimate that full generic competition will take place in 2022 based on previous trends of generic competition. Our study found significant heterogeneity in the magnitude of savings associated with generic entry, which is consistent with prior studies.^24,25^

### Barriers and Incentives to Generic Entry

Brand-name manufactures have an incentive to engage in strategies to delay the introduction of generic drugs and reduce competition.^26^ Policies to encourage generic entry, such as the 1984 Hatch-Waxman Act, can promote lower prices.^21,22,27,28^ For instance, the total annual expenditure for atorvastatin rose in 2016 (Figure 2) because of an increase in the manufacturer’s price; in 2017, however, the annual expenditure decreased after a drop in the manufacturer’s price because of the competition from additional generics.

### Generic Competition Among Different Major Payers

Generic competition has had different impacts across payers, some of which are able to negotiate prices.^29^ The current study found that generic entry was associated with a 98.6% reduction in Medicare spending on statins, which is higher than that estimated in prior studies.^30,31^ Crestor was among the top 3 most costly drugs for Medicare Part D in 2015, costing the program $3 billion, according to Centers for Medicare & Medicaid Services data.^11^ The period with 1 brand and 1 generic competitor was associated with an 85.5% decrease in Medicare’s Crestor spending, a finding consistent with other independent estimates.^31^ With additional generic entry, expenditures declined another 7.6%. Medicare’s relatively high savings after generic statin availability is partially due to the relatively high price that the program was paying for brand-name statins. Under Medicare Part D, the Centers for Medicare & Medicaid Services are prohibited from negotiating drug prices and exercising supervision over drug companies.^32^ The introduction of Part D in 2006 caused a shift in prescription drug expenditures from Medicaid to Medicare (Figure 3).^33^ The observed increase in Medicare spending on brand-name and generic statins is reflective of this shift.

Medicaid programs experienced modest cost savings after the introduction of generic competition. Possible reasons for the relatively smaller reduction include the price spike observed in 20% of generic drugs covered under Medicaid between 2014 and 2017^34^ as well as other structural barriers.^35^ Our cost savings estimates for Medicaid are likely conservative because the rebates received by payers (ranging from 45% to 76% between 2015 and 2018) are unaccounted for.^36,37^ The complicated rebate system embedded in pharmacy benefit managers could also have resulted in conservative national estimates of the private health insurance cost savings.^38^ Private insurers incurred the highest savings, a result possibly attributable to the generic competition in commercial health plans and high OOP spending.^39^

Our findings on reductions in OOP expenditures are comparable to other estimates. These relatively large savings likely partially reflect a relatively steep brand-name statin list price previously faced by cash-paying patients who held little bargaining power compared with large private and public insurers, as well as the trend toward high deductibles among the privately insured. In light of the evidence that people with lower incomes purchase generic medications more often,^40^ generic competition reduces disparities and increases access to affordable medication. However, the mean duration of market exclusivity of statins is 13.1 years, a time period that greatly exceeds other market exclusivity federal statutory minimums, such as the 5- to 7-year minimum for small molecules, and perpetuates disparities in access.^4,41^ Such long market exclusivity durations and their drug expenditure implications may not be optimal for all US patients.^42,43^

### Limitations

This study had several limitations. First, although the Medical Expenditure Panel Survey uses a Medical Provider Component to validate the number of purchases and associated spending, the possibility of nonresponse bias remains. Second, the Multum Lexicon Code procedures to identify statins as either branded or generic may have led to coding errors. Unexpected fluctuations are present, for example, in the number of purchases and expenditures of Zocor (Figure 1 and Figure 2). Nevertheless, we decided to not modify the original data owing to lack of sufficient information. Third, the cost-saving estimates for Medicaid are likely conservative because the analysis did not account for rebates. Fourth, the influence of other newly available treatment options was not evaluated. For instance, the introduction of ezetimibe and proprotein convertase subtilisin/kexin type 9 (PSCK9) inhibitors as adjuvant therapy has shown promising treatment benefits when compared with statin monotherapy.^31,44,45^ Nevertheless, their effect on expenditures of brand-name and generic statins is not fully understood. The clinical use of such agents remains limited given their current high cost.

## Conclusions

This survey study found that full generic competition of statins was associated with significant cost savings across all major payers within the US health care system, saving $925.60 for individual US statin users annually and $11.9 billion nationwide. The encouragement of full generic competition and early access to generic medication options should be prioritized in future regulatory actions.

## Notes

Supplement. **eAppendix 1.** Study Design **eAppendix 2.** Statin Use Trends **eFigure 1.** Sample Selection **eFigure 2.** Trends of Annual Total and Out-of-Pocket Expenditures for Selected Statins Before and After Patent Expiration **eFigure 3.** Trends of Insurer Spending on Low-Moderate–Intensity Statins Before and After Patent Expiration **eFigure 4.** Trends of Medicaid Spending on High-Intensity Statins Before and After Patent Expiration **eFigure 5.** Parallel Trend Assumption Check—Brand-Name Statins Number of Purchases **eFigure 6.** Parallel Trend Assumption Check—Brand-Name Statins Total Annual Expenditures **eFigure 7.** Parallel Trend Assumption Check—Generic Statins Number of Purchases **eFigure 8.** Parallel Trend Assumption Check—Generic Statins Total Annual Expenditures **eTable 1.** The Modified Park Test **eTable 2.** Association Between End of Market Exclusivity and Number of Statin Purchases and Expenditures per Panel DID Estimates—Subgroup Analysis **eTable 3.** Annual Number of Purchases of All Prescription Drugs and Statins **eTable 4.** AAPOR Standard Disclosure Form
