# Supplementary material for: Trends in Use and Expenditures for Brand-name Statins After Introduction of Generic Statins in the US, 2002-2018
Source: JAMA Netw Open. 2021 Nov 22;4(11):e2135371. doi: 10.1001/jamanetworkopen.2021.35371 (PMC8609409; doi:10.1001/jamanetworkopen.2021.35371)
Supplement: Supplement. — eAppendix 1. Study Design eAppendix 2. Statin Use Trends eFigure 1. Sample Selection eFigure 2. Trends of Annual Total and Out-of-Pocket Expenditures for Selected Statins Before and After Patent Expiration eFigure 3. Trends of Insurer Spending on Low-Moderate–Intensity Statins Before and After Patent Expiration eFigure 4. Trends of Medicaid Spending on High-Intensity Statins Before and After Patent Expiration eFigure 5. Parallel Trend Assumption Check—Brand-Name Statins Number of Purchases eFigure 6. Parallel Trend Assumption Check—Brand-Name Statins Total Annual Expenditures eFigure 7. Parallel Trend Assumption Check—Generic Statins Number of Purchases eFigure 8. Parallel Trend Assumption Check—Generic Statins Total Annual Expenditures eTable 1. The Modified Park Test eTable 2. Association Between End of Market Exclusivity and Number of Statin Purchases and Expenditures per Panel DID Estimates—Subgroup Analysis eTable 3. Annual Number of Purchases of All Prescription Drugs and Statins eTable 4. AAPOR Standard Disclosure Form [file jamanetwopen-e2135371-s001.pdf]

**Supplementary Online Content**

Lin SY, Baumann K, Zhou C, Zhou W, Cuellar AE, Xue H. Trends in use and expenditures for brand-name statins after introduction of generic statins in the US, 2002-2018. *JAMA Netw Open*. 2021;4(11):e2135371. doi:10.1001/jamanetworkopen.2021.35371

**eAppendix 1.** Study Design
**eAppendix 2.** Statin Use Trends
**eFigure 1.** Sample Selection
**eFigure 2.** Trends of Annual Total and Out-of-Pocket Expenditures for Selected Statins Before and After Patent Expiration
**eFigure 3.** Trends of Insurer Spending on Low-Moderate–Intensity Statins Before and After Patent Expiration
**eFigure 4.** Trends of Medicaid Spending on High-Intensity Statins Before and After Patent Expiration
**eFigure 5.** Parallel Trend Assumption Check—Brand-Name Statins Number of Purchases
**eFigure 6.** Parallel Trend Assumption Check—Brand-Name Statins Total Annual Expenditures
**eFigure 7.** Parallel Trend Assumption Check—Generic Statins Number of Purchases
**eFigure 8.** Parallel Trend Assumption Check—Generic Statins Total Annual Expenditures
**eTable 1.** The Modified Park Test
**eTable 2.** Association Between End of Market Exclusivity and Number of Statin Purchases and Expenditures per Panel DID Estimates—Subgroup Analysis
**eTable 3.** Annual Number of Purchases of All Prescription Drugs and Statins
**eTable 4.** AAPOR Standard Disclosure Form

This supplementary material has been provided by the authors to give readers additional information about their work.

© 2021 Lin SY et al. *JAMA Network Open.*

**eAppendix 1.** Study Design

**Study design**
Seven statins were included in the study: Lipitor, Crestor, Zocor, Pravachol, Mevacor, Lescol, and Livalo. Five generic statins were available as of 2018 and included: Atorvastatin (generic form of Lipitor), Rosuvastatin (generic form of Crestor), Simvastatin (generic form of Zocor), Lovastatin (generic form of Mevacor), and Pravastatin (generic form of Pravachol).

**Panel DID**
The first D referred to the difference in the number of purchases and expenditures of each brand-name statin before and after patent expiration. The second D referred to the difference between a given brand-name statin that set to end of its market exclusivity in a given year and all other brand-name statins with market exclusivity yet to end prior to that given year. For instance, the market exclusivity of Lipitor ended in 2011, the comparison group for Lipitor includes Crestor (market exclusivity ended in 2016), Lescol (market exclusivity ended in 2020) and Livalo (market exclusivity ended in 2020).

**The construction of outcome variables**
In the present study, we included total annual expenditure, payment by private insurance, Medicaid, Medicare and out-of-pocket. Although expenditures were self-reported in MEPS, MEPS used Medical Provider Component (MPC) as one of the main sources of information about expenditures and key to the validity of the measures. MPC is a survey of hospitals, physicians, pharmacies, and other medical facilities that provided care to sampled household members in the course of the survey year. Patients in sampled households were asked to sign permission forms authorizing contact with their health care providers. MPC was used to supplement information provided by household survey respondents to fill in missing expenditures. Per capita aggregate costs were presented in total. Total healthcare expenditures reflected the sum of 12 source of payment categories for each reported medical encounter in the survey—including out of pocket by patient, Medicare, Medicaid, Private insurance, Veteran's administration, TRICARE, other Federal Sources, other State or local sources, worker's compensation, unclassified sources, other private, and other public. The cost estimates were adjusted to 2018 U.S. dollar (USD) based on CPI-PMED (The price index for prescription drugs). For missing expenditures, MEPS used predictive mean matching imputation to impute missing values. This procedure used regression models to predict total expenses for each event. For each event with missing expenditure, a donor event with closest predicted payment with the same pattern of expected payment sources was used to impute the missing expenditure.

**Data Processes and the implementation of sampling weight**
The single full year person-level weight was used. The weighting process for each panel included adjustments for nonresponse over time and a calibration to independent population totals. The detail construct of sampling weight can be found in the MEPS methodology report.[1] The strata and PSUs were used for the variance estimation.[1] First, we merged prescription drug event files with full year consolidation files to obtain the number of statin purchases and corresponding expenditures at the individual level (n=58,354). This included statins purchased by insurers as well as out-of-pocket purchases. The files included demographic attributes as well as the patient's past medical history. Second, we calculated a representative annual number of purchases and expenditures using sampling weights along with strata and PSUs at individual

© 2021 Lin SY et al. *JAMA Network Open.*

level. All other demographic characteristics and prevalence of medical conditions were converted to percentages or averages. For each class of statin, we converted individual annual purchases and expenditures into nationally representative estimates by applying sampling weights. A final analytical panel data structure with nationally representative estimates were then created for each statin class. A final analytical panel data structure with nationally representative estimates were then created based on statin class (n=204). To be more specific, the weighted summary statistics presented in table 1 were weighted at individual level first and then converted to population level. The detail calculation formula is presented below.

**Proportion**
For demographic characteristics we included in the study, the annualized proportion was reported,

$$Summary\ statistics\ of\ covariates\ for\ each\ statin\ class = \frac{\left(\sum_{j=1}^{17} \frac{(\sum_{i=1}^{n} X \times weight_i)}{(weighted\ total\ population)_j}\right)}{17}$$

Where X denotes to each covariates (past medical history, access to care and so forth). Weight denotes to the single full year person-level weight for individual who responded to MEPS for the full period of time that he or she was in-scope during the survey year. The detail development of person-level weight can be found in the MEPS methodology report. (https://meps.ahrq.gov/data_files/publications/mr24/mr24.pdf). Weighted total population represented the total population in a given year. J denotes to our observational period (from 2002 to 2018, or 17 years).

The summary statistics are the annualized population average. Take female as an example to illustrate. We first calculated the proportion of female population in a given year, and averaged them by a factor of 17 (observational period).

**Sum**
For the number of purchases and expenditures, the annualized sum was reported,

$$\#\ of\ purchases\ and\ expenditures\ of\ each\ statin\ class = \frac{\left(\sum_{j=1}^{17} \sum_{i=1}^{n} X \times weight_i\right)}{17}$$

Where X denotes to an individual’s annual number of statin purchases and expenditures. Weight denotes to the single full year person-level weight for individual who responded to MEPS for the full period of time that he or she was in-scope during the survey year. The detail development of person-level weight can be found in the MEPS methodology report. (https://meps.ahrq.gov/data_files/publications/mr24/mr24.pdf). J denotes to our observational period (from 2002 to 2018, or 17 years).

The summary statistics are the annualized population sum total annual expenditure. Using Lipitor as an example to illustrate, we first calculated the national total expenditure of Lipitor for each given year, and averaged them by a factor of 17 (observational period).

© 2021 Lin SY et al. *JAMA Network Open.*

**eAppendix 2.** Statin Use Trends

**Statin Use Trends**

As shown in Figure 1, following patent expiration of Zocor in 2005, annual prescriptions decreased by 72%—from 38.3 million to 10.7 million in two years—and remained below 1.8 million per year after 2011. After its 2001 patent expiration, the annual number of generic lovastatin increased sixfold from 3.86 million in 2002 to 21.1 million in 2007. The number of purchased decreased to 8.76 million by the end of 2018. Similarly, the number of generic pravastatin purchases surged almost tenfold after Pravachol went off patent in 2006 from 3.22 million to 35 million in 2012. This surge was followed by a downtrend to 24 million in 2018.

**Spending Trends for High-Intensity Statins**

As shown in the figure 3, the annual Medicaid spending on Lipitor peaked at $1.5 billion in 2004. After Medicare launched in 2006, Medicaid spending on Lipitor decreased to $0.37 billion in 2007, further shrank to $0.04 billion in 2013, and stayed below $0.1 billion by the end of 2018. The Medicaid expenditure for generic atorvastatin increased for the first four years, decreased to $0.29 billion in 2016, and remained below $0.4 billion as of 2018.

**Total Annual Expenditures**

**Atorvastatin**

After patent expiration in 2011, the total annual expenditure for Lipitor dropped to $4.02 billion in 2012 and $0.48 billion in 2013. Following a moderate increase to $1.45 billion in 2016, spending on Lipitor decreased by 96.5% to $0.05 billion in 2018. Total annual expenditure for generic atorvastatin rose to $4.02 billion in 2012, gradually increased to $5.76 in 2014, and stabilized at around $6 billion throughout 2018.

**Simvastatin**

Meanwhile, annual expenditure for generic rosuvastatin sharply increased. The annual expenditure for Zocor fell by more than 87% to $1.04 billion in 2007 and subsequently remained below $1 billion. Annual expenditure for generic simvastatin reached its highest level at $7.34 billion in 2008 and gradually decreased until 2017 when the total stabilized at $1.5 billion.

© 2021 Lin SY et al. *JAMA Network Open.*

**eFigure 1.** Sample Selection

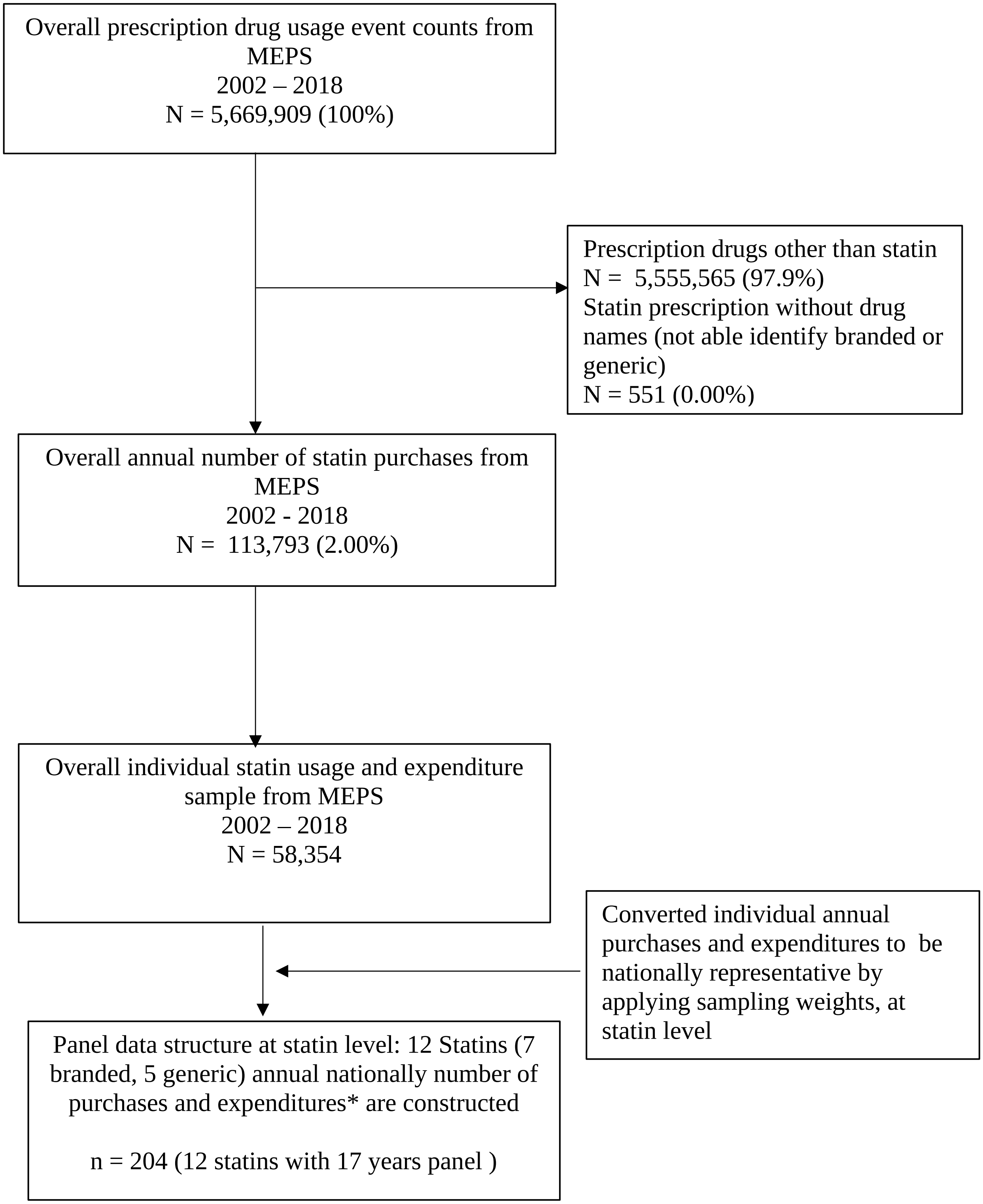


Notes: * for some years no statin use was reported, we imputed a small number (i.e. 100) to replace 0 for better model convergence

© 2021 Lin SY et al. *JAMA Network Open.*

**eFigure 2.** Trends of Annual Total and Out-of-Pocket Expenditures for Selected Statins Before and After Patent Expiration

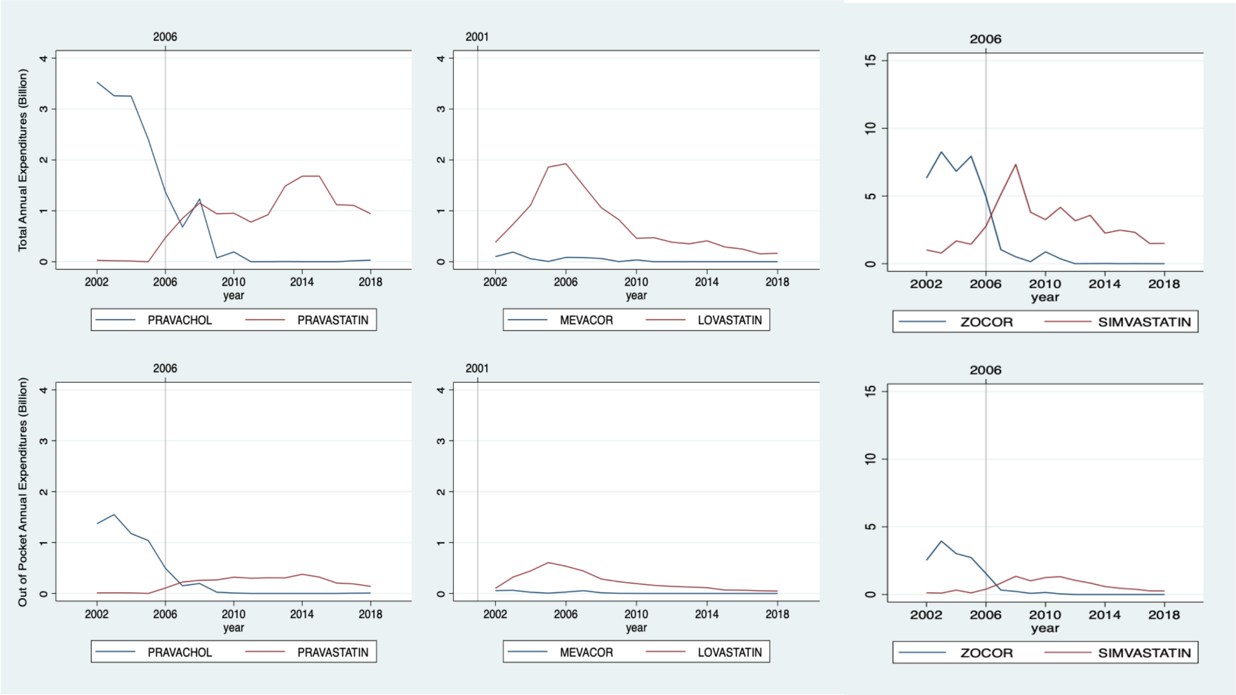


Notes: The vertical gray line indicates the year of patent expiration. The blue line represents the trend of brand-name statin, and red line represents its generic counterpart. All expenditures in billions of dollars, 2018.

**eFigure 2**

**Out-of-pocket expenditures**

The market exclusivity of Mevacor, one statin that can be used as low to moderate intensity, ended in 2001. Since 2002, the annual out-of-pocket (OOP) spending on Mevacor was no larger than $0.06 billion and was close to zero after 2009. On the other hand, the OOP of generic lovastatin surged sixfold between 2002 and 2005, from $0.1 billion to $0.61 billion. After 2006, when Zocor went off patent, the OOP of this substitute statin declined through the end of 2018, to $0.05 billion in 2018. The OOP of Pravachol, another low to moderate intensity, peaked in 2003 at $1.55 billions of annual spending, but decreased significantly afterward. After the end of market exclusivity in 2006, the annual OOP further dropped to $0.03 billion in 2009 and reached close to zero between 2011 and 2018.

**Total annual expenditures**

Total expenditures of Mevacor were under $0.2 billion between 2002 and 2018. Total expenditures of generic lovastatin increased from $0.38 billion to $1.93 billion in 2006, but sharply decreased as the generic simvastatin entered the market. After 2010, the total annual spending on generic lovastatin was below $0.5 billion and decreased further in 2018 to $0.17 billion. With the end of market exclusivity in 2006 total expenditures of Pravachol decreased from $3.53 billion in 2002 to $0.19 in 2010 and approached zero after 2011.

© 2021 Lin SY et al. *JAMA Network Open.*

**eFigure 3.** Trends of Insurer Spending on Low-Moderate–Intensity Statins Before and After Patent Expiration

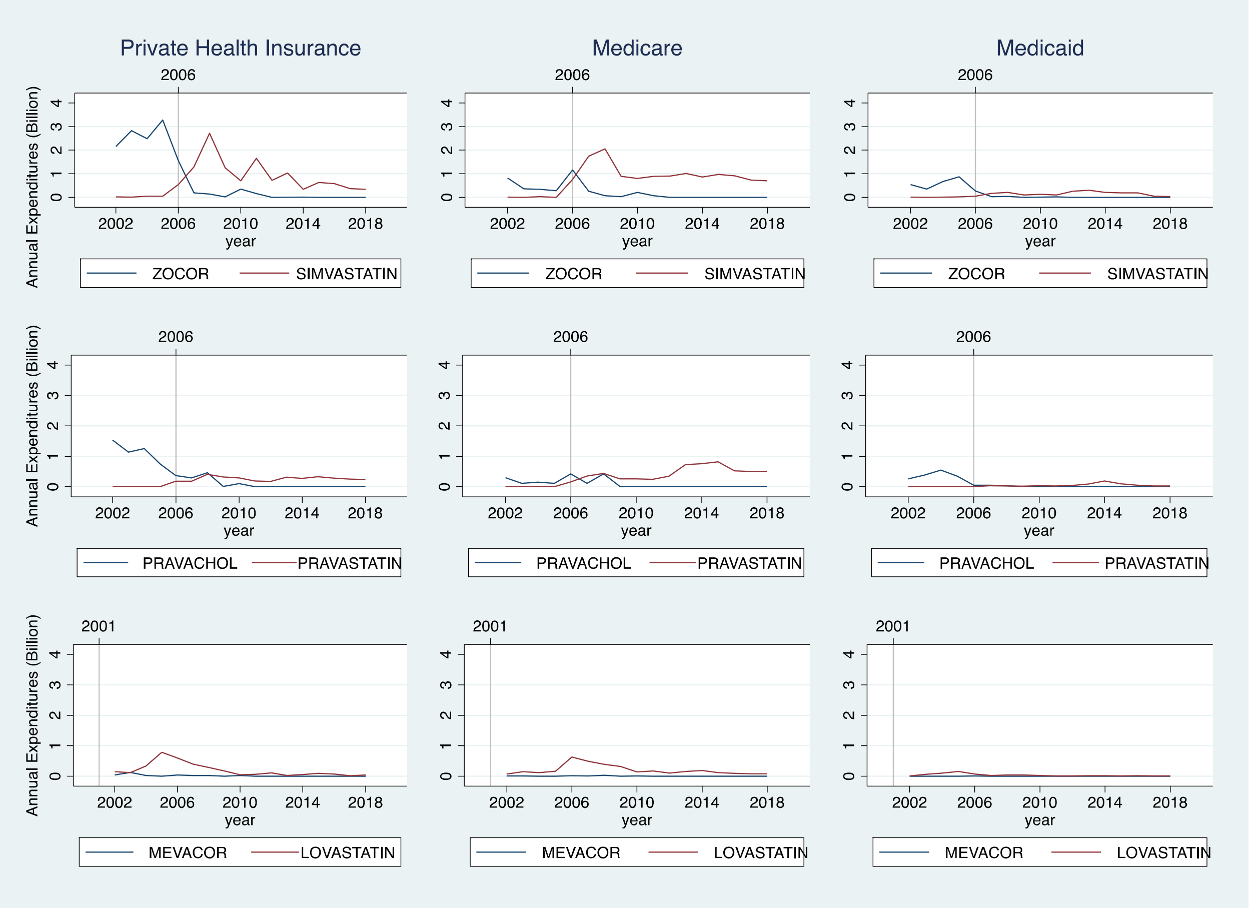


Notes: The vertical gray line indicates the year of patent expiration. The blue line represents the trend of brand-name statin, and red line represents its generic counterparts. All Billion are presented in 2018 USD

**eFigure 3**

**Trends in payments by major payors**

**Private**

Private insur expenditures for low to moderate intensity statins (simvastatin, lovastatin and pravastatin) are shown in eFigure2. After the end of market exclusivity, the annual expenditures for Zocor by private insurers dropped from $3.28 billion in 2005 to $0.19 billion in 2007. In 2018, the annual generic simvastatin spending by private health insurance was $0.34 billion. Since the end of market exclusivity , the annual private health insurance spending on Mevacor was below $0.12 billion. The generic lovastatin, climbed to $0.79 billion in 2005 and fell sharply after 2010. The spending on Pravachol declined from $1.53 billion in 2002 to $0.01 billion in 2009 and remained below $0.01 billion at the end of 2018. Private health insurance expenditures for generic pravastatin declined from $0.32 billion to $0.17 billion between 2009 and 2018.

**Medicaid**

Medicaid expenditures for low to moderate intensity statins (simvastatin, lovastatin and pravastatin) are shown in eFigure2. After the end of market exclusivity, expenditures for Zocor declined from $0.87 billion in 2005 to $0.03 billion in 2007.

Medicaid spending on generic simvastatin increased moderately after 2006, but declined to $0.03 billion in 2018. Similar trends were observed in Mevacor. Medicaid spending on generic pravastatin did not change significantly increased after 2011 when Affordable Care Act (ACA) was implemented. The Medicaid annual spending on generic pravastatin dropped from $0.19 billion in 2014 to $0.03 billion in 2018, close to the amount spent before 2011.

**Medicare**

Medicare expenditures for low to moderate intensity statins (simvastatin, lovastatin and pravastatin) are shown in figure S2. Notably, the end of market exclusivity for Zocor coincided with the Medicare Part D implementation in 2006. As a result, there was a jump of Medicare Zocor spending in 2006, from $0.28 billion in 2005 to $1.16 billion. However, the Medicare annual spending of Zocor quickly declined to $0.03 in 2009 and remained low afterward.

© 2021 Lin SY et al. *JAMA Network Open.*

**eFigure 4.** Trends of Medicaid Spending on High-Intensity Statins Before and After Patent Expiration

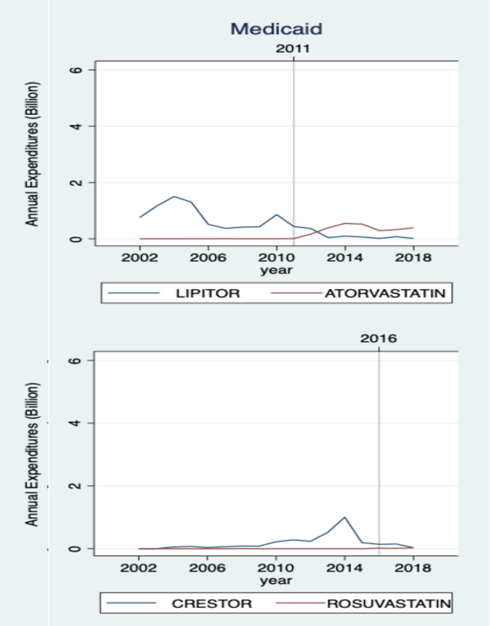


Notes: The vertical gray line indicates the year of patent expiration. The blue line represents the trend of brand-name statin, and red line represents its generic counterparts. All Billion are presented in 2018 USD

**eFigure 5.** Parallel Trend Assumption Check—Brand-Name Statins Number of Purchases

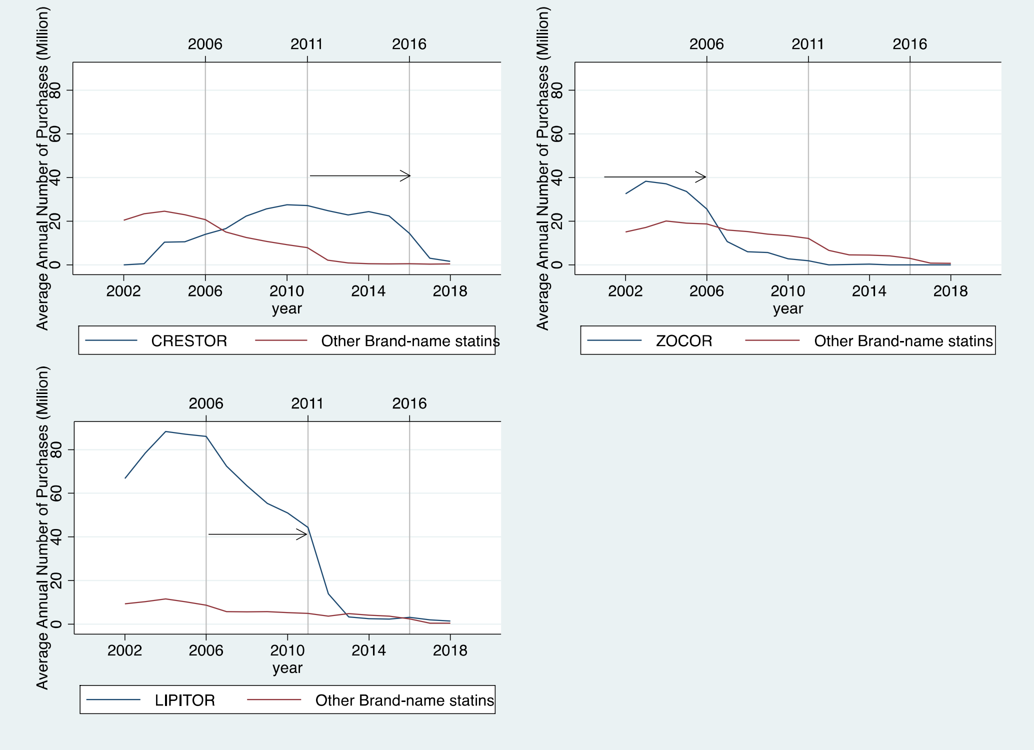


Notes: The vertical gray lines indicate the year of patent expiration. The blue line represents the trend of brand-name statin, and red line represents other brand-name counterparts. The solid arrow denotes to the period testing of parallel trend assumption.

© 2021 Lin SY et al. *JAMA Network Open.*

Parallel trends assumption check: Using Zocor as an example, from the figure S4 we observed that prior to 2006 (the year Zocor's market exclusivity ended), the average annual number of purchases between Zocor and other available generic statins had similar trends: both increased from 2002 to 2004 and decreased afterwards through 2006.

© 2021 Lin SY et al. *JAMA Network Open.*

**eFigure 6.** Parallel Trend Assumption Check—Brand-Name Statins Total Annual Expenditures

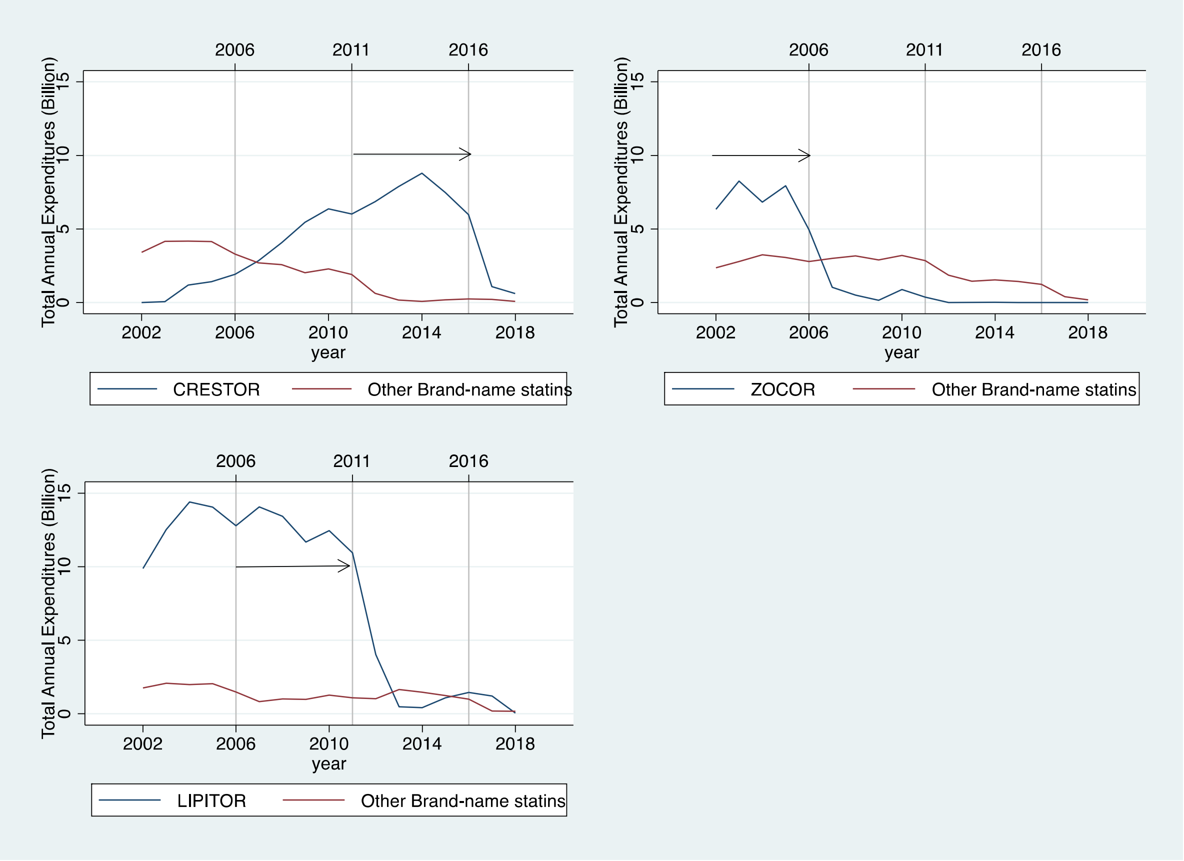


Notes: The vertical gray lines indicate the year of patent expiration. The blue line represents the trend of brand-name statin, and red line represents other brand-name counterparts. The solid arrow denotes to the period testing of parallel trend assumption.

© 2021 Lin SY et al. *JAMA Network Open.*

**eFigure 7.** Parallel Trend Assumption Check—Generic Statins Number of Purchases

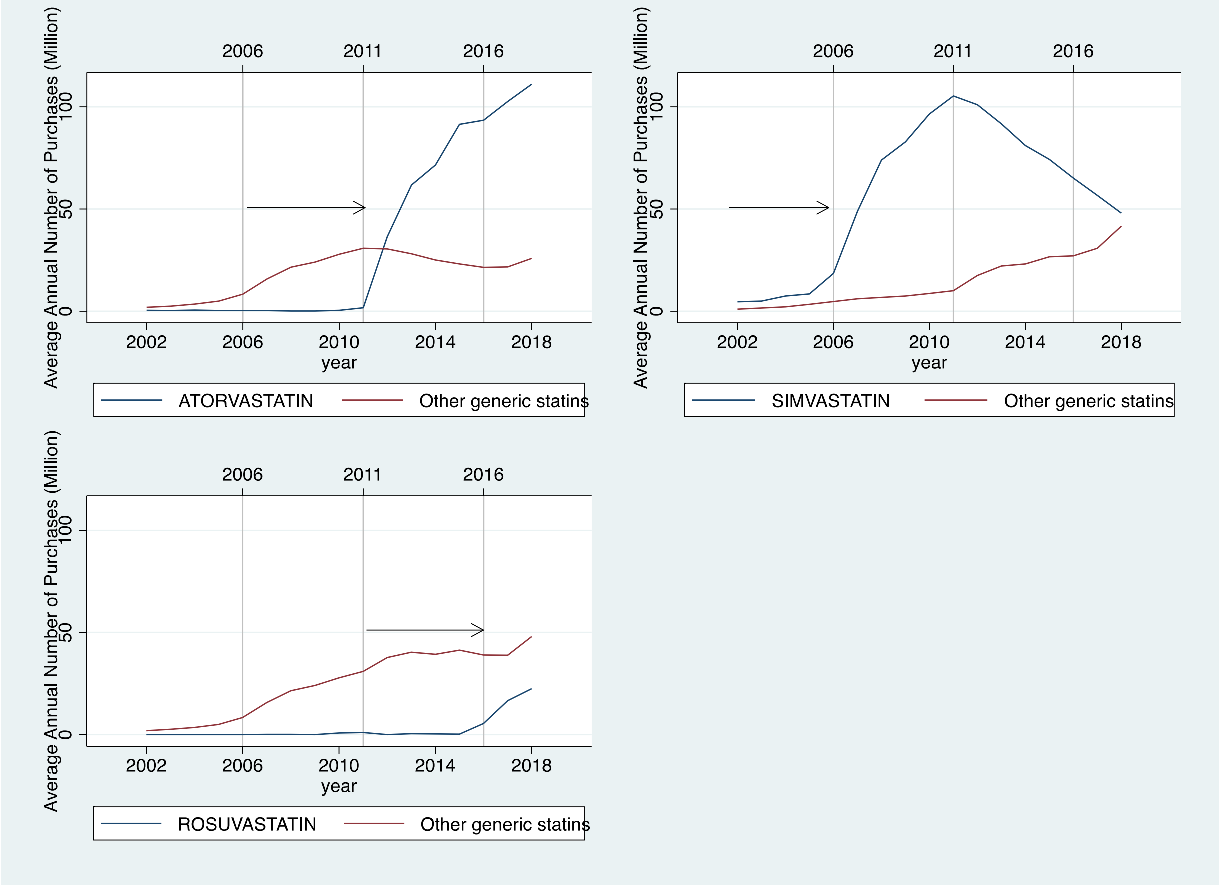


Notes: The vertical gray lines indicate the year of patent expiration. The blue line represents the trend of generic statin, and red line represents other generic counterparts. The solid arrow denotes to the period testing of parallel trend assumption.

© 2021 Lin SY et al. *JAMA Network Open.*

**eFigure 8.** Parallel Trend Assumption Check—Generic Statins Total Annual Expenditures

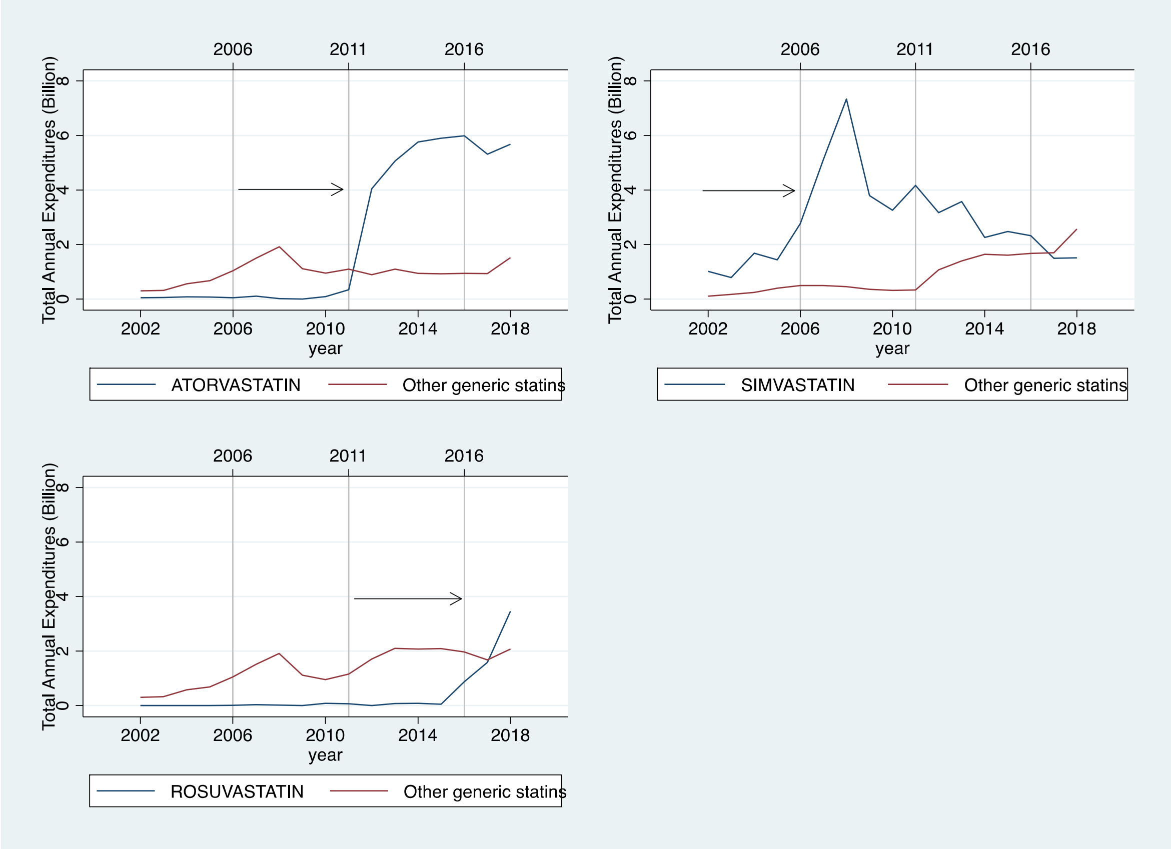


Notes: The vertical gray lines indicate the year of patent expiration. The blue line represents the trend of generic statin, and red line represents other generic counterparts. The solid arrow denotes to the period testing of parallel trend assumption.

© 2021 Lin SY et al. *JAMA Network Open.*

**eTable 1.** The Modified Park Test

| family of distribution | F statistic | p-value |
|---|---|---|
| **Generic drugs** | | |
| Gaussian | 1121.89 | 0 |
| Poisson | 301.46 | 0 |
| Gamma* | 1.52 | 0.2184 |
| Inverse Gaussian | 222.04 | 0 |
| **Brand name drugs** | | |
| Gaussian | 347.52 | 0 |
| Poisson | 85.92 | 0 |
| Gamma* | 0.01 | 0.9175 |
| Inverse Gaussian | 89.80 | 0 |

*The gamma distribution is chosen in fitting the GLM model because it fails to reject the null hypothesis, suggesting the expenditure follows the gamma distribution.

© 2021 Lin SY et al. *JAMA Network Open.*

**eTable 2.** Association Between End of Market Exclusivity and Number of Statin Purchases and Expenditures per Panel DID Estimates— Subgroup Analysis

| | Number of purchases† | Private insurance § | Medicare§ | Medicaid§ | Out-of-pocket§ | Total annual expenditure§ |
|---|---|---|---|---|---|---|
| | IRR | Effect£ | Effect£ | Effect£ | Effect£ | Effect£ |
| **Individual level** | | | | | | |
| **Prevention indications**[∂] | | | | | | |
| Primary | 0.736** | -400.4*** | -261.4*** | -69.12*** | -206.6*** | -926.8*** |
| | (0.569 - 0.951) | (-468.3 - -332.4) | (-327.1 - -195.8) | (-96.34 - -41.90) | (-226.7 - -186.4) | (-1,013 - -840.3) |
| *Interpretation* | 26.4% decrease | $400.4 reduction | $261.4 reduction | $69.12 reduction | $206.6 reduction | $826.8 reduction |
| Secondary | 0.716*** | -246.2 | -259.7*** | -162.5*** | -257.6 | -835.6*** |
| | (0.561 - 0.914) | (-295.6 - -196.7) | (-317.9 - -201.5) | (-277.7 - -47.28) | (-299.0 - -216.1) | (-908.2 - -762.9) |
| *Interpretation* | 28.4% decrease | $246.2 reduction | $259.7 reduction | $162.5 reduction | $257.6 reduction | $835.6 reduction |
| **Race/Ethnicity** | | | | | | |
| Asian | 0.74 | -1,157*** | -310*** | -323.4 | -201.7*** | -1,287*** |
| | (0.502 - 1.089) | (-1,950 - -364.2) | (-598.6 - -22.09) | (-659.1 - 12.19) | (-253.6 - -149.9) | (-1,635 - -939.6) |
| *Interpretation* | 26% decrease | $1,157 reduction | $310 reduction | $323.4 reduction | $201.7 reduction | $1,287 reduction |
| Black/African American | 0.524 | -258.9*** | -384.7 | -178.5*** | -155.7*** | -889.1*** |
| | (0.245 - 1.118) | (-323.0 - -194.8) | (-1,378 - 608.9) | (-277.5 - -79.47) | (-201.6 - -109.8) | (-1,041 - -737.0) |
| *Interpretation* | 47.6% decrease | $258.9 reduction | $384.7 reduction | $178.5 reduction | $155.7 reduction | $889.1 reduction |
| White | 0.751*** | -378.9*** | -329.0*** | -60.95*** | -222.2*** | -936.0*** |
| | (0.612 - 0.922) | (-449.0 - -308.9) | (-453.0 - -204.9) | (-82.86 - -39.04) | (-244.6 - -199.8) | (-1,025 - -847.5) |
| *Interpretation* | 24.9% decrease | $378.9 reduction | $329 reduction | $60.95 reduction | $222.2 reduction | $936 reduction |

**∂:** The prevention indication was defined based on the history of coronary heart disease, heart attack (myocardial infraction), stable angina and stroke. We defined a patient receiving statin therapy for secondary prevention with the confirmed diagnosis of any one of these four indications.

† Number of purchases (presented in incidence rate ratio, IRR) was estimated by negative binomial model, controlling for female, white, black, Asian, married, public health insurance, having usual source of care, past medical history (stroke, heart attack, CHD, angina), and age

§ Individual-level expenditure was estimated by GLM model, gamma distribution with log link function, controlling percent of female, married, public health insurance, having usual source of care, past medical history (stroke, heart attack, CHD, angina) and average age

*** $p < 0.01$, ** $p < 0.05$, 95% CI presented in parentheses.

© 2021 Lin SY et al. *JAMA Network Open.*

eTable 2

Secondary prevention for statin therapy is defined by ASCVD, which includes acute coronary syndrome, stable angina, arterial revascularization, cerebral vascular accident (stroke), transient ischemic attack (TIA), and peripheral artery disease (PAD). In the study, we captured four main secondary prevention indications—history of coronary artery disease, myocardial infarction (MI), stable angina, and stroke. We characterized statin therapy with an existence of any one of these four conditions as secondary prevention. Statin therapy without the existence of any one of the four conditions was characterized as primary prevention. We acknowledge that this labeling method does not perfectly characterize statin use as primary or secondary prevention. Nevertheless, since the four included indications capture the bulk of ASCVD in the United States, we are confident mislabeling within our study is minimal. Furthermore, the sample population had an average age of 68.6 years old (SE: 0.14 years old) and a diabetes prevalence of 29.4%. This prevalence estimate is comparable to the rates among US population reported by the American Diabetes Association (ADA).[2] Therefore, we are confident our subgroup analysis captures the majority of primary prevention statin usage.

The results of the subgroup analysis suggested that the end of market exclusivity was significantly correlated with a decrease in average number of purchases of brand-name statins for primary- and secondary- prevention indications (26.4% and 28.4% respectively). The cost saving incurred by private insurance for primary prevention was higher than secondary prevention ($400.4 reduction vs. $246.2 reduction). On the other hand, the cost saving for secondary prevention ($162.5 reduction, [95% CI] = -277.7 - -47.28) was greater for Medicaid than primary prevention ($69.12 reduction, [95% CI] = -96.34 - -41.90). Overall, the total cost saving for each individual incurred by the end of statins' market exclusivity was greater for primary prevention indications ($926.8 [95% CI] = -1,013 - -840.3 annual reduction in comparison with $835.6 reduction [95% CI] = -908.2 - -762.9).

In terms of the race/ethnicity-based subgroup analysis, we observed different patterns of cost saving after the end of market exclusivity. The end of market exclusivity was associated with a 47.6% ([95% CI] = 10% -75%) decrease in the number of brand-name statins purchases for African Americans, which was higher than the number of purchases within the Asian (26% decrease [95% CI] = 5% - 43%) and white (24.9% decrease [95% CI] = 8% - 39%) population. The end of market exclusivity was associated with $1,157 ([95% CI] = -1,950 - - 364.2) of cost savings for the Asian population, which was higher than the number of purchases within the African American/Black ($258.9 cost savings, [95% CI] = -323.0 - -194.8) and White population ($378.9 cost savings, [95% CI] = -449.0 - -308.9). The pattern for out-of-pocket cost savings were similar. Overall, the cost savings were highest for the Asian population followed by the White and African American/Black population.

© 2021 Lin SY et al. *JAMA Network Open.*

**eTable 3.** Annual Number of Purchases of All Prescription Drugs and Statins

| Year | Sample size of all prescription drugs purchased | Annual number of statin purchases | Individuals who were prescribed statins |
|---|---|---|---|
| 2002 | 354813 | 15494 | 2594 |
| 2003 | 318064 | 15288 | 2461 |
| 2004 | 331423 | 17460 | 2878 |
| 2005 | 331774 | 17449 | 2914 |
| 2006 | 356197 | 19351 | 3092 |
| 2007 | 313138 | 17630 | 2944 |
| 2008 | 307850 | 18566 | 3061 |
| 2009 | 349686 | 21224 | 3601 |
| 2010 | 316030 | 20402 | 3436 |
| 2011 | 329892 | 21554 | 3694 |
| 2012 | 343287 | 22348 | 3896 |
| 2013 | 344531 | 21917 | 3844 |
| 2014 | 335379 | 20046 | 3658 |
| 2015 | 346276 | 21335 | 3886 |
| 2016 | 335327 | 20933 | 3946 |
| 2017 | 324260 | 20799 | 4070 |
| 2018 | 331982 | 21253 | 4379 |
| Total | 5669909 | 114344 | 58354 |

© 2021 Lin SY et al. *JAMA Network Open.*

**eTable 4.** AAPOR Standard Disclosure Form

| AAPOR STANDARD DISCLOSURE FORM | |
|---|---|
| **BASIC DISCLOSURE ELEMENTS** | **DETAILS** |
| Survey sponsor | Agency for Healthcare Research and Quality |
| Survey/Data collection supplier | Agency for Healthcare Research and Quality |
| Population represented | Non-institutionalized families and individuals in the United States |
| Sample size | Around three thousand per-annum for 17 years, of total 58,354 |
| Mode of data collection | Computer assisted personal interview (CAPI) in English |
| Type of sample (probability/non-probability) | Probability |
| Start and end dates of data collection | 2002 – 2018 annually |
| Margin of sampling error for total sample | See MEPS methodology report for more details[1] |
| Margin of sampling error for key subgroups | See MEPS methodology report for more details[1] |
| Are the data weighted? | Yes, to national targets from the Current Population Survey (CPS) |
| Contact for more information | Agency for Healthcare Research and Quality<br><br>5600 Fishers Lane<br>Rockville, MD 20857<br>Telephone: (301) 427-1364 |
| Survey questions are publicly available in the official website of the Department of Health & Human Services https://www.meps.ahrq.gov/mepsweb/ | |

© 2021 Lin SY et al. *JAMA Network Open.*

**eReferences**


1. Ezzati-Rice TM, Rohde F, Greenblatt J. Methodology Report# 22: Sample Design of the Medical Expenditure Panel Survey Household Component, 1998–2007. In:2009.
2. American Diabetes A. Economic Costs of Diabetes in the U.S. in 2017. *Diabetes Care.* 2018;41(5):917.


© 2021 Lin SY et al. *JAMA Network Open.*
